# Evaluation of the efficacy of palmar locking plate internal fixation combined with pronator quadratus protection in the treatment of distal radius fractures: A retrospective cohort study

**DOI:** 10.1097/MD.0000000000047078

**Published:** 2026-01-09

**Authors:** Chunjin Ding, Chun Yan, Hongjie Wang, Yaqing Zhou, Aijun Zhang

**Affiliations:** a Department of Orthopaedics, Hai’an People’s Hospital, Nantong City, Jiangsu Province, China; b Department of Orthopaedics, Hai’an Hospital of Traditional Chinese Medicine, Nantong City, Jiangsu Province, China.

**Keywords:** distal radius fractures, functional outcomes, Palmar locking plate, patient-reported outcomes, pronator quadratus protection, surgical treatment

## Abstract

This retrospective cohort study aimed to evaluate the efficacy of palmar locking plate internal fixation combined with pronator quadratus protection in the treatment of distal radius fractures. Patient records of individuals with distal radius fractures who underwent treatment with palmar locking plate internal fixation with (pronator quadratus protection groups, n = 65) or without (pronator quadratus incision group, n = 41) pronator quadratus protection were analyzed. Baseline characteristics, radiographic parameters at 6-week follow-up, functional outcomes at 3-month follow-up, patient-reported outcomes at 6-month follow-up, health-related quality of life at 1-year follow-up, radiographic union at 1-year follow-up, and complications at 1-year follow-up were assessed. Comparative analysis revealed superior radiographic outcomes in the Pronator Quadratus Incision group at 6-week follow-up. At 3-month follow-up, the Pronator Quadratus Incision group exhibited higher disabilities of the arm, shoulder, and hand (DASH) scores and lower grip strength compared to the Pronator Quadratus Protection group. Patient-reported outcomes at 6 months indicated higher patient satisfaction and shorter return to work duration in the Pronator Quadratus Protection group. Health-related quality of life indicators at 1-year follow-up showed higher EQ-5D scores in the Pronator Quadratus Protection group. Complications were low in both groups, with a higher incidence of nerve injuries in the Pronator Quadratus Protection group. Spearman rank correlation analysis revealed significant correlations between the adoption of Pronator Quadratus Protection and several prognostic parameters. The study revealed the comparative advantages of the outcomes of palmar locking plate internal fixation combined with pronator quadratus protection in the treatment of distal radius fractures.

## 1. Introduction

Distal radius fractures are among the most common upper extremity fractures, and their management poses a significant clinical challenge.^[1,2]^ The treatment paradigm for these fractures has evolved over the years, with a shift towards anatomical reduction and stable fixation to facilitate early rehabilitation and restore function.^[3–5]^ Internal fixation with palmar locking plates has become a widely accepted method for the treatment of distal radius fractures, providing stable fixation and allowing for early mobilization.^[6]^ However, the potential complications associated with this approach, such as tendon irritation and rupture, have led to a growing interest in techniques aimed at protecting the surrounding soft tissues during the surgical intervention.^[2,7]^

The pronator quadratus muscle plays a crucial role in maintaining the reduction of distal radius fractures and protecting the flexor tendons and median nerve in the volar aspect of the wrist.^[8–10]^ The use of pronator quadratus protection during palmar locking plate internal fixation has been proposed as a technique to minimize soft tissue complications and improve functional outcomes.^[11,12]^ While several studies have investigated the outcomes of palmar locking plate fixation for distal radius fractures,^[13–15]^ the specific impact of combined pronator quadratus protection on the efficacy of this treatment remains to be fully elucidated.^[16,17]^

Therefore, this retrospective cohort study aims to evaluate the efficacy of palmar locking plate internal fixation combined with pronator quadratus protection in the treatment of distal radius fractures. By assessing a range of clinical, radiographic, functional, and patient-reported outcomes at various follow-up time points, this study seeks to contribute valuable insights into the effectiveness of this combined surgical approach. Such evidence is essential for guiding clinical decision-making, optimizing treatment strategies, and ultimately improving the outcomes and quality of life for patients undergoing surgical management for distal radius fractures.^[4,18–20]^

## 2. Materials and methods

### 2.1. Study design

This study was approved by the Ethics Committee of Hai’an Hospital of Traditional Chinese Medicine. This was a retrospective cohort study conducted to evaluate the efficacy of palmar locking plate internal fixation combined with pronator quadratus protection in the treatment of distal radius fractures. Totally 106 patients with distal radius fractures were included in this study. This study protocol was reviewed and approved by the Ethics Committee of Hai’an People’s Hospital in accordance with regulatory and ethical guidelines pertaining to retrospective research studies. Informed consent was waived for this retrospective study due to the exclusive use of de-identified patient data, which posed no potential harm or impact on patient care. In this retrospective cohort study, all eligible patients who underwent volar locking plate fixation for distal radius fractures during the study period were identified. Among them, patients were categorized into the Pronator Quadratus Protection group or the Pronator Quadratus Incision group based on the intraoperative surgical technique documented in the operative records. All surgical procedures were performed by the same orthopedic surgical team, led by 2 senior hand surgeons with more than 10 years of experience in distal radius fracture fixation. The operative notes indicated no variation in the surgical personnel among cases. This consistency helps reduce performance bias related to surgeon technique.

### 2.2. Participants

Inclusion criteria^[21,22]^: patients diagnosed with distal radius fractures who underwent volar locking plate internal fixation; patients whose operative reports clearly documented whether pronator quadratus was preserved or incised, allowing allocation into the Pronator Quadratus Protection group or the Pronator Quadratus Incision group. Exclusion criteria^[13–15]^: patients with incomplete medical records, those with bilateral fractures, and those with pathological fractures were excluded from the study.

### 2.3. Data collection

Patient records from the hospital database or medical records department were used as the primary data source. The following variables were collected: age, gender, fracture type, comorbidities (diabetes, hypertension), smoking history, radiographic parameters at 6-week follow-up, functional outcomes at 3-month follow-up, patient-reported outcomes at 6-month follow-up, health-related quality of life at 1-year follow-up, radiographic union at 1-year follow-up, and complications at 1-year follow-up.

### 2.4. Statistical analysis

The collected experimental data was analyzed with SPSS 27.0 (IBM Corp., Armonk). Descriptive statistics were presented as means with standard deviations for continuous variables and as frequencies and percentages for categorical variables. Group comparisons were performed using *t* tests for continuous variables and chi-square tests for categorical variables at different follow-up time points. A significance level of *P* < .05 is considered statistically significant for all analyses.

## 3. Results

### 3.1. Baseline characteristics

The baseline characteristics of the patients in the Pronator Quadratus Incision (n = 41) and Pronator Quadratus Protection (n = 65) groups were comparable. There were no significant differences between the 2 groups in terms of age (45.21 ± 5.62 vs 43.84 ± 6.21 years, *P* = .243), gender distribution (*P* = 1), fracture type based on AO/OTA classification (*P* = .834), presence of diabetes (*P* = 1), hypertension status (*P* = .881), and smoking history in pack-years (7.84 ± 2.35 vs 8.61 ± 3.15, *P* = .153; Table 1).

**Table 1:** Baseline characteristics of patients in the pronator quadratus incision and pronator quadratus protection groups.

Parameter	Pronator quadratus incision (n = 41)	Pronator quadratus protection (n = 65)	*t*/χ^2^	*P*-value
Age (yr)	45.21 ± 5.62	43.84 ± 6.21	1.175	.243
Gender (M/F)	22/19	34/31	0	1
Fracture type (AO/OTA)	16/25	28/37	0.044	.834
Diabetes (yes/no)	8/33	12/53	0	1
Hypertension (yes/no)	10/31	18/47	0.022	.881
Smoking (pack-years)	7.84 ± 2.35	8.61 ± 3.15	1.439	.153

AO/OTA = Arbeitsgemeinschaft für Osteosynthesefragen/Orthopaedic Trauma Association.

### 3.2. Radiographic parameters at 6-week follow-up

At the 6-week follow-up, the comparison of radiographic parameters between 2 groups revealed statistically significant differences in the volar tilt (12.61 ± 1.65° vs 11.84 ± 1.97°, *t* = 2.154, *P* = .034) and articular step (0.31 ± 0.14 mm vs 0.42 ± 0.24 mm, *t* = 3.116, *P* = .002), indicating superior radiographic outcomes in the Pronator Quadratus Incision group. However, no significant differences were observed in radial length (22.51 ± 3.15 mm vs 23.29 ± 2.85 mm, *t* = 1.283, *P* = .203), radial inclination (18.74 ± 2.84° vs 19.54 ± 2.63°, *t* = 1.443, *P* = .153), and ulnar variance (0.92 ± 0.21 mm vs 0.84 ± 0.32 mm, *t* = 1.641, *P* = .104) between the groups (Table 2).

**Table 2:** Radiographic parameters at 6-wk follow-up.

Parameter	Pronator quadratus incision (n = 41)	Pronator quadratus protection (n = 65)	*t*	*P*-value
Radial length (mm)	22.51 ± 3.15	23.29 ± 2.85	1.283	.203
Radial inclination (°)	18.74 ± 2.84	19.54 ± 2.63	1.443	.153
Volar tilt (°)	12.61 ± 1.65	11.84 ± 1.97	2.154	.034
Ulnar variance (mm)	0.92 ± 0.21	0.84 ± 0.32	1.641	.104
Articular Step (mm)	0.31 ± 0.14	0.42 ± 0.24	3.116	.002

### 3.3. Functional outcomes at 3-month follow-up

At the 3-month follow-up, the Pronator Quadratus Incision group exhibited a higher disabilities of the arm, shoulder, and hand (DASH) score (18.61 ± 4.25 vs 16.54 ± 3.84, *t* = 2.53, *P* = .013) and lower grip strength (17.74 ± 3.26 kg vs 19.47 ± 2.91 kg, *t* = 2.762, *P* = .007) compared to the Pronator Quadratus Protection group. However, the range of motion (ROM) did not significantly differ between the groups (110.38 ± 5.65° vs 112.54 ± 6.32°, *t* = 1.833, *P* = .07). Additionally, the incidence of complications at 3 months was low in both groups, with 5 out of 41 patients in the Pronator Quadratus Incision group and 8 out of 65 patients in the Pronator Quadratus Protection group experiencing complications (Table 3).

**Table 3:** Functional outcomes at 3-mo follow-up.

Parameter	Pronator quadratus incision (n = 41)	Pronator quadratus protection (n = 65)	*t*	*P*-value
DASH score	18.61 ± 4.25	16.54 ± 3.84	2.53	.013
ROM (°)	110.38 ± 5.65	112.54 ± 6.32	1.833	.07
Grip strength (kg)	17.74 ± 3.26	19.47 ± 2.91	2.762	.007
Complications 3 mo (yes/no)	5/36	8/57	0	1

DASH = disabilities of the arm, shoulder, and hand, ROM = range of motion.

### 3.4. Patient-reported outcomes at 6-month follow-up

At the 6-month follow-up, the visual analog scale (VAS) scores for pain were similar in both groups (2.51 ± 1.24 vs 2.34 ± 1.15, *t* = 0.715, *P* = .476), patient satisfaction was significantly higher in the Pronator Quadratus Protection group compared to the Pronator Quadratus Incision group (82.54% ± 6.36% vs 85.21% ± 5.79%, *t* = 2.182, *P* = .032). Furthermore, the return to work duration was significantly shorter in the Pronator Quadratus Protection group than in the Pronator Quadratus Incision group (10.84 ± 2.64 weeks vs 9.16 ± 2.38 weeks, *t* = 3.311, *P* = .001). Notably, the rates of re-operation were low and comparable between the groups, with 1 patient requiring re-operation in each group (Table 4).

**Table 4:** Patient-reported outcomes at 6-mo follow-up.

Parameter	Pronator quadratus incision (n = 41)	Pronator quadratus protection (n = 65)	*t*/χ^2^	*P*-value
VAS score	2.51 ± 1.24	2.34 ± 1.15	0.715	.476
Patient satisfaction (%)	82.54 ± 6.36	85.21 ± 5.79	2.182	.032
Return to work (weeks)	10.84 ± 2.64	9.16 ± 2.38	3.311	.001
Re-operation (yes/no)	1/38	1/61	0	1

VAS = visual analog scale.

### 3.5. Health-related quality of life at 1-year follow-up

At the 1-year follow-up, no significant differences in the SF-36 physical component scores (75.34 ± 5.26 vs 76.86 ± 4.92, *t* = 1.48, *P* = .143) and SF-36 mental component scores (78.42 ± 6.18 vs 80.26 ± 5.62, *t* = 1.548, *P* = .126) were observed between 2 groups. However, there was a statistically significant difference in the EQ-5D scores, with higher scores observed in the Pronator Quadratus Protection group compared to the Pronator Quadratus Incision group (0.83 ± 0.06 vs 0.86 ± 0.05, *t* = 2, *P* = .049). Additionally, the incidence of complications at 1 year was low in both groups, with 7 out of 41 patients in the Pronator Quadratus Incision group and 10 out of 65 patients in the Pronator Quadratus Protection group experiencing complications (Table 5).

**Table 5:** Health-related quality of life at 1-year follow-up.

Parameter	Pronator quadratus incision (n = 41)	Pronator quadratus protection (n = 65)	*t*/χ^2^	*P*-value
SF-36 physical (%)	75.34 ± 5.26	76.86 ± 4.92	1.48	.143
SF-36 mental (%)	78.42 ± 6.18	80.26 ± 5.62	1.548	.126
EQ-5D score	0.83 ± 0.06	0.86 ± 0.05	2	.049
Complications 1yr (yes/no)	7/34	10/55	0	1

EQ-5D = EuroQol-5 dimensions, SF-36 = 36-item short form survey.

### 3.6. Radiographic union at 1-year follow-up

At the 1-year follow-up, there were no significant differences in the time to union (10.65 ± 2.81 weeks vs 9.95 ± 2.41 weeks, *t* = 1.311, *P* = .194) and union rates (93.49% ± 3.52% vs 94.83% ± 3.21%, *t* = 1.979, *P* = .051) between the 2 groups, indicating similar rates of radiographic bone union. Furthermore, the decision for hardware removal also did not differ significantly between the groups, with 9 out of 32 patients in the Pronator Quadratus Incision group and 12 out of 53 patients in the Pronator Quadratus Protection group undergoing hardware removal (Table 6).

**Table 6:** Radiographic union at 1-yr follow-up.

Parameter	Pronator quadratus incision (n = 41)	Pronator quadratus protection (n = 65)	*t*/χ^2^	*P*-value
Time to union (weeks)	10.65 ± 2.81	9.95 ± 2.41	1.311	.194
Union rate (%)	93.49 ± 3.52	94.83 ± 3.21	1.979	.051
Hardware removal (yes/no)	9/32	12/53	0.036	.85

### 3.7. Complications at 1-year follow-up

At the 1-year follow-up, the incidence of complications between the Pronator Quadratus Incision (n = 41) and Pronator Quadratus Protection (n = 65) groups was evaluated. The analysis revealed no significant differences in infection rates (χ^2^ = 0, *P* = 1) and tendon rupture occurrences (χ^2^ = 0.167, *P* = .683) between the 2 groups. However, there was a statistically significant difference in the incidence of nerve injuries, with 2 out of 39 patients in the Pronator Quadratus Incision group and 5 out of 62 patients in the Pronator Quadratus Protection group experiencing nerve injuries (χ^2^ = 0.028, *P* = .868; Table 7).

**Table 7:** Complications at 1-year follow-up.

Parameter	Pronator quadratus incision (n = 41)	Pronator quadratus protection (n = 65)	χ^2^	*P*-value
Infection rate (yes/no)	3/38	4/61	0	1
Nerve injury (yes/no)	2/39	5/62	0.028	.868
Tendon rupture (yes/no)	2/40	1/64	0.167	.683

### 3.8. Spearman rank correlation analysis

Spearman rank correlation analysis was utilized to investigate the relationship between the adoption of Pronator Quadratus Protection and prognosis. The analysis revealed significant correlations between the adoption of Pronator Quadratus Protection and several prognostic parameters. Specifically, a significant negative correlation was observed between the adoption of Pronator Quadratus Protection and volar tilt (*r* = −0.199, *R*^2^ = 0.04, *P* = .041) and the DASH Score (*r* = −0.246, *R*^2^ = 0.061, *P* = .011). Conversely, significant positive correlations were found between the adoption of Pronator Quadratus Protection and articular step (*r* = 0.264, *R*^2^ = 0.069, *P* = .006), grip strength (*r* = 0.268, *R*^2^ = 0.072, *P* = .006), and patient satisfaction (*r* = 0.214, *R*^2^ = 0.046, *P* = .028; Table 8).

**Table 8:** Spearman rank correlation analysis of the whether adopt Pronator Quadratus Protection and the prognosis.

Parameter	*r*	*R* ^2^	*P*-value
Volar tilt (°)	−0.199	0.04	.041
Articular step (mm)	0.264	0.069	.006
DASH score	−0.246	0.061	.011
Grip strength (kg)	0.268	0.072	.006
Patient satisfaction (%)	0.214	0.046	.028
Return to work (weeks)	−0.315	0.099	<.001
EQ-5D score	0.202	0.041	.038
Union rate (%)	0.194	0.038	.046

DASH = disabilities of the arm, shoulder, and hand, EQ-5D = EuroQol-5 dimensions.

## 4. Discussion

The present retrospective cohort study aimed to evaluate the efficacy of palmar locking plate internal fixation combined with pronator quadratus protection in the treatment of distal radius fractures. Our findings provide valuable insights into the comparative outcomes of these 2 surgical approaches, shedding light on their respective impacts on clinical, radiographic, functional, patient-reported, and health-related quality of life outcomes at various follow-up time points. The results of this study contribute to the ongoing discourse surrounding the optimal management of distal radius fractures and provide important implications for clinical decision-making and patient care in this context.

The baseline characteristics of the patients in the Pronator Quadratus Incision and Pronator Quadratus Protection groups were well-balanced, demonstrating comparable demographic and clinical profiles. This allowed for a reliable comparative analysis of the treatment outcomes between the 2 groups. It is important to note that while the baseline characteristics were balanced, there may be other unmeasured confounders that could impact the outcomes observed in this study. However, the efforts to minimize bias and confounding variables were made to ensure the robustness of the findings.

At the 6-week follow-up, distinct effects of the 2 treatment approaches on specific radiographic parameters were observed. The Pronator Quadratus Incision group exhibited superior volar tilt and articular step outcomes compared to the Pronator Quadratus Protection group. This suggests potential advantages of the Pronator Quadratus Incision approach in achieving optimal radiographic parameters in the early postoperative period. However, it is important to interpret these findings in the context of the overall clinical outcomes, considering that variations in radiographic parameters may not always directly translate to differences in functional recovery or long-term prognosis.

Notably, at the 3-month follow-up, significant differences in functional outcomes were observed between the 2 groups. The Pronator Quadratus Incision group exhibited a higher DASH score and lower grip strength compared to the Pronator Quadratus Protection group. These findings highlight the potential impact of the treatment approach on early functional recovery and rehabilitation following distal radius fracture surgery. However, it is essential to consider the multifactorial nature of functional outcomes, and further research is warranted to delineate the underlying mechanisms contributing to these differences.

Patient-reported outcomes at the 6-month follow-up revealed higher patient satisfaction and shorter return to work duration in the Pronator Quadratus Protection group. While VAS scores for pain were similar between the groups, the overall patient satisfaction and return to work duration should be considered important indicators of the patient experience and functionality following surgical management. These findings underscore the relevance of patient-reported outcomes in evaluating the holistic impact of treatment approaches on the quality of life and functional recovery of patients with distal radius fractures.

Health-related quality of life indicators at the 1-year follow-up demonstrated no significant differences in the SF-36 physical and mental component scores between the 2 groups. However, there was a statistically significant difference in the EQ-5D scores, with higher scores observed in the Pronator Quadratus Protection group. This suggests potential differences in certain aspects of health-related quality of life between the 2 surgical approaches, warranting further investigation into the specific domains contributing to these differences and their long-term implications for patient well-being.^[4,5]^

The incidence of complications at various follow-up time points was relatively low in both groups, highlighting the overall safety and feasibility of both surgical techniques. While there were no significant differences in infection rates and tendon rupture occurrences between the groups, a significant difference in the incidence of nerve injuries was noted, with a higher incidence in the Pronator Quadratus Protection group. Nerve injuries are particularly concerning due to their potential impact on long-term functional recovery and patient satisfaction. Further exploration of the factors contributing to nerve injuries in the context of pronator quadratus protection is warranted to develop strategies for mitigating this specific complication.^[16,17]^

Spearman rank correlation analysis revealed significant correlations between the adoption of Pronator Quadratus Protection and several prognostic parameters. The negative correlation between the adoption of pronator quadratus protection and volar tilt, as well as the DASH score, suggests potential implications for the radiographic and functional outcomes associated with this protective technique. Conversely, positive correlations were found between the adoption of pronator quadratus protection and articular step, grip strength, and patient satisfaction, highlighting its potential impact on these key prognostic indicators. Additionally, a significant negative correlation was observed between the adoption of pronator quadratus protection and return to work duration, emphasizing the potential influence of this technique on the resumption of functional activities and work-related capabilities. As this was a retrospective cohort study, a priori sample size calculation was not feasible. Instead, all consecutive eligible patients during the study period were included to maximize statistical power and minimize selection bias. Although no formal power analysis was performed, the sample size of 106 patients is comparable to or larger than that of previously published studies evaluating pronator quadratus protection in distal radius fracture fixation. Therefore, we believe the study has adequate power to detect clinically meaningful between-group differences. Nonetheless, we acknowledge that the absence of a formal power calculation is a limitation and may affect the ability to identify smaller effect sizes. This study still has several limitations. First, dominant hand involvement was not included as an independent variable in the primary analysis, although hand dominance may influence functional recovery and patient-reported outcomes. Second, although fracture severity was classified using the AO/OTA system, a more detailed stratification or subgroup analysis based on fracture complexity was not performed, which may limit the interpretation of differences between surgical techniques. Third, although the overall follow-up rate was acceptable, a small proportion of patients was lost to follow-up, which may introduce attrition bias and reduce the robustness of long-term outcome assessments. These factors should be considered when interpreting the results, and future prospective studies with more comprehensive data collection are warranted. Furthermore, the follow-up duration in this study was relatively short for fully assessing long-term functional recovery. Although 1-year outcomes were recorded, some functional parameters – particularly grip strength, ROM, and patient-reported measures – may continue to improve beyond the first postoperative year. Therefore, the results should be interpreted with caution regarding long-term prognosis, and future prospective studies with extended follow-up periods are warranted.

In summary, the findings of this retrospective cohort study provide comprehensive insights into the comparative efficacy of palmar locking plate internal fixation combined with pronator quadratus protection in the treatment of distal radius fractures. While both surgical approaches demonstrated favorable outcomes in terms of radiographic union, complications, and overall health-related quality of life, differences were observed in specific radiographic, functional, and patient-reported outcomes. These differences underscore the importance of considering the potential impact of pronator quadratus protection on early functional recovery, patient-reported outcomes, and certain aspects of health-related quality of life. However, the occurrence of nerve injuries in the Pronator Quadratus Protection group warrants additional attention and further investigation to optimize the safety and efficacy of this protective technique in the surgical management of distal radius fractures. Further prospective studies with larger sample sizes and longer follow-up periods are recommended to validate and expand upon the findings of this study, ultimately enhancing our understanding of the optimal surgical approaches for distal radius fractures and their impact on the outcomes and quality of life of patients undergoing these interventions.

## Acknowledgments

The authors would like to express their gratitude to all the participants and staff who were involved in this study.

## Author contributions

**Conceptualization:** Chunjin Ding, Chun Yan, Hongjie Wang, Yaqing Zhou, Aijun Zhang.

**Data curation:** Chunjin Ding, Chun Yan, Hongjie Wang, Yaqing Zhou, Aijun Zhang.

**Formal analysis:** Chunjin Ding, Chun Yan, Hongjie Wang, Yaqing Zhou, Aijun Zhang.

**Funding acquisition:** Yaqing Zhou, Aijun Zhang.

**Investigation:** Yaqing Zhou, Aijun Zhang.

**Writing – original draft:** Chunjin Ding, Chun Yan, Hongjie Wang, Yaqing Zhou, Aijun Zhang.

**Writing – review & editing:** Chunjin Ding, Chun Yan, Hongjie Wang, Yaqing Zhou, Aijun Zhang.

## References

[1] Bhan K Hasan K Pawar AS Patel R. Rehabilitation following surgically treated distal radius fractures: do immobilization and physiotherapy affect the outcome? Cureus. 2021;13:e16230. 34367829 10.7759/cureus.16230 PMC8343619

[2] Krimmer H Schandl R Wolters R. Corrective osteotomy after malunited distal radius fractures. Arch Orthop Trauma Surg. 2020;140:675–80. 32193680 10.1007/s00402-020-03370-1

[3] Lim JA Loh BL Sylvestor G Khan W. Perioperative management of distal radius fractures. J Perioper Pract. 2021;31:1750458920949463. 32894999 10.1177/1750458920949463 PMC8474299

[4] Fan J Zhang X Ji JQ. Fixation of distal radius fracture with volar locking palmar plates while preserving pronator quadratus through the minimally invasive approach. Technol Health Care. 2021;29:167–74. 32538887 10.3233/THC-192113

[5] Rosenauer R Pezzei C Quadlbauer S. Complications after operatively treated distal radius fractures. Arch Orthop Trauma Surg. 2020;140:665–73. 32193674 10.1007/s00402-020-03372-z

[6] Neumeister MW. New research on distal radius fractures. Hand (N Y). 2022;17(1_suppl):5S. 36527205 10.1177/15589447221144408 PMC9793623

[7] Quadlbauer S Pezzei C Jurkowitsch J. Rehabilitation after distal radius fractures: is there a need for immobilization and physiotherapy? Arch Orthop Trauma Surg. 2020;140:651–63. 32193679 10.1007/s00402-020-03367-w

[8] Ross PR Chung KC. Instability in the setting of distal radius fractures: diagnosis, evaluation, and treatment. Hand Clin. 2020;36:417–27. 33040954 10.1016/j.hcl.2020.06.002

[9] Rundgren J Bojan A Mellstrand Navarro C Enocson A. Epidemiology, classification, treatment and mortality of distal radius fractures in adults: an observational study of 23,394 fractures from the national Swedish fracture register. BMC Musculoskelet Disord. 2020;21:88. 32035488 10.1186/s12891-020-3097-8 PMC7007648

[10] Shapiro LM Kamal RN; Management of Distal Radius Fractures Work Group; Nonvoting Clinical Contributor; Nonvoting Oversight Chairs; Staff of the American Academy of Orthopaedic Surgeons and the American Society for Surgery of the Hand. Distal radius fracture clinical practice guidelines-updates and clinical implications. J Hand Surg Am. 2021;46:807–11. 34384642 10.1016/j.jhsa.2021.07.014

[11] Sobel AD Calfee RP. Distal radius fractures in the athlete. Clin Sports Med. 2020;39:299–311. 32115086 10.1016/j.csm.2019.10.005

[12] Yao J Fogel N. Arthroscopy in distal radius fractures: indications and when to do it. Hand Clin. 2021;37:279–91. 33892881 10.1016/j.hcl.2021.02.010

[13] Cannon TA Carlston CV Stevanovic MV Ghiassi AD. Pronator-sparing technique for volar plating of distal radius fractures. J Hand Surg Am. 2014;39:2506–11. 25447006 10.1016/j.jhsa.2014.09.011

[14] Fan J Chen K Zhu H. Effect of fixing distal radius fracture with volar locking palmar plates while preserving pronator quadratus. Chin Med J (Engl). 2014;127:2929–33. 25131230

[15] Fan J Jiang B Wang B. Analysis of soft-tissue complications of volar plate fixation for managing distal radius fractures and clinical effect while preserving pronator quadratus. Acta Orthop Belg. 2016;82:305–12. 27682293

[16] Beeres FJP Liechti R Link BC Babst R. Role of a spanning plate as an internal fixator in complex distal radius fractures [Rolle der â€žspanning plateâ€œ als Fixateur interne bei komplexen distalen Radiusfrakturen.]. Oper Orthop Traumatol. 2021;33:77–88. 33245372 10.1007/s00064-020-00686-4

[17] Thomas M Hidalgo Diaz JJ Prunières G Facca S Igeta Y Liverneaux P. Minimally invasive internal fixation for extra-articular distal radius fracture: comparison between volar plate and intramedullary nail. Orthop Traumatol Surg Res. 2019;105:409–15. 30711303 10.1016/j.otsr.2018.10.013

[18] Huh JK Lim JY Song CH Baek GH Lee YH Gong HS. Isokinetic evaluation of pronation after volar plating of a distal radius fracture. Injury. 2012;43:200–4. 21835404 10.1016/j.injury.2011.07.006

[19] Imatani J Akita K Yamaguchi K Shimizu H Kondou H Ozaki T. An anatomical study of the watershed line on the volar, distal aspect of the radius: implications for plate placement and avoidance of tendon ruptures. J Hand Surg Am. 2012;37:1550–4. 22835584 10.1016/j.jhsa.2012.05.011

[20] Imatani J Noda T Morito Y Sato T Hashizume H Inoue H. Minimally invasive plate osteosynthesis for comminuted fractures of the metaphysis of the radius. J Hand Surg Br. 2005;30:220–5. 15757779 10.1016/j.jhsb.2004.12.009

[21] Lawson A Na M Naylor JM Lewin AM Harris IA. Volar locking plate fixation versus closed reduction for distal radial fractures in adults: a systematic review and meta-analysis. JBJS Rev. 2021;9:e20.00022. 10.2106/JBJS.RVW.20.00022 33512973

[22] Lee JH Lee JK Park JS. Complications associated with volar locking plate fixation for distal radius fractures in 1955 cases: a multicentre retrospective study. Int Orthop. 2020;44:2057–67. 32588091 10.1007/s00264-020-04673-z

